# Chronotype, Time of Day, and Performance on Intelligence Tests in the School Setting

**DOI:** 10.3390/jintelligence11010013

**Published:** 2023-01-11

**Authors:** Konrad S. Jankowski, Juan Francisco Díaz-Morales, Christian Vollmer

**Affiliations:** 1 Department of Psychology, University of Warsaw, 00-183 Warsaw, Poland; 2 Department of Social Psychology, Work and Individual Differences, Faculty of Psychology, Complutense University of Madrid, 28223 Madrid, Spain; 3 Department of Research and Development in Teacher Education, University College of Teacher Education Tyrol, 6020 Innsbruck, Austria

**Keywords:** chronotype, morningness–eveningness, fluid intelligence, crystallized intelligence, high school

## Abstract

Research suggests the existence of an association between chronotype and intellectual performance, but the nature of this link remains unclear. Studies conducted in a laboratory setting point to the *synchrony effect* (better performance at a person’s preferred time of day) for fluid intelligence, but not for crystallized intelligence, whereas studies that have analyzed students’ grades suggest that the effect exists for both. In the present study, we aimed to verify the synchrony effect by applying direct measures of crystallized intelligence, fluid intelligence, and subjective sleepiness–alertness in a sample of high school students during their morning or afternoon class. The results revealed a synchrony effect for crystallized, but not for fluid intelligence. During morning class, students with a morning chronotype performed better than evening chronotypes on a test of crystallized intelligence, whereas during afternoon class there was no difference between chronotypes. The association resulted from decreased performance during morning class in evening chronotypes that improved during afternoon class and constant performance in morning chronotypes. These effects were independent of sleepiness–alertness levels. The results suggest that individual differences between chronotypes may be important for tasks performed during morning classes, but not during afternoon ones, and that performance across school days may depend on time of day in evening chronotypes.

## 1. Introduction

The term *chronotype*, also called the *morningness–eveningness dimension*, describes individual differences in the preferred and actual timing for sleep and activity (Jankowski 2013). Morning chronotypes function and sleep during earlier clock times than evening chronotypes, and this time shift has been observed in many domains (Bailey and Heitkemper 2001). Chronotype has biological underpinnings, with genetic factors explaining around 50% of variability (Koskenvuo et al. 2007), but it is also influenced by environment, with light being the proven direct agent (Porcheret et al. 2018).

The association between chronotype and intellectual performance is a matter of dispute, and various conflicting outcomes can be found in the literature. An initial study that suggested that evening chronotypes are more intelligent than morning chronotypes was conducted on a sample biased toward morningness: military recruits after six weeks of preparatory training that imposed on them a homogeneous sleep–wake cycle (Roberts and Kyllonen 1999). In that study, two indicators of intelligence were used. At random times of the day, the Armed Services Vocational Aptitude Battery was administered to the participants. This battery can be viewed as a measure of crystallized intelligence because it relies on acquired knowledge about general science, arithmetic reasoning, word knowledge, paragraph comprehension, numerical operations, coding speed, auto and shop information, mathematics, mechanical comprehension, and electrical information. Another measure in the study, which was administered between 8:00 and 12:00, was composed of subtests assessing more basic cognitive processes: working memory, processing speed, and estimating clock time. The results indicated that eveningness was related to better performance in processing speed and working memory. It should be noticed that working memory can be treated as a proxy for fluid intelligence (Au et al. 2015).

Results indicating higher fluid intelligence in evening chronotypes compared with morning ones were also found in an experimental study that directly assessed fluid intelligence in a convenience sample of adults tested during afternoon hours (but another study in this series did not replicate the outcome; Zajenkowski et al. 2019). The above-mentioned series of two studies did not reveal associations between chronotype and crystallized intelligence (Zajenkowski et al. 2019). Results regarding fluid intelligence were further supported by a different study, of high-achieving graduate students, showing that evening chronotypes scored higher on measures of general intelligence than morning ones (Piffer et al. 2014). We must note, however, that some research has shown no associations between chronotype and general intelligence (e.g., as indicated by the comparison of morningness–eveningness levels between Mensa members versus matched nonmembers, Ujma et al. 2020).

A somewhat different picture emerges from studies conducted in school settings, which have indicated that morning chronotypes, compared with evening ones, not only obtain higher scores in fluid intelligence but also get higher academic grades (Arbabi et al. 2015), which can be considered a proxy for crystallized intelligence (Zhang and Ziegler 2022). Associations of higher academic performance with morningness have appeared consistently across various studies, but they can be offset by delaying school day starts that cancel the academic supremacy of morning chronotypes (Goldin et al. 2020). Such an improvement in performance by evening chronotypes attending delayed school day shifts indicates a possible synchrony effect in school settings.

The term *synchrony effect* describes an expectation that, in principle, chronotypes perform best at their preferred time of day. The effect was initially observed in word problem-solving tasks completed by young adults (evening chronotypes) and older adults (morning chronotypes), who were compared at their presumed peak (8:00 for morning chronotypes) and off-peak (17:00 for morning chronotypes) times of day (May 1999). The most striking observation was that performance in older adults during their peak times was similar to that obtained by young adults during their off-peak times, suggesting that the synchrony effect can help to make up for age-related cognitive decline.

The occurrence of the synchrony effect in intelligence tests among school students has also been tested. Goldstein et al. (2007) conducted their study in a laboratory setting, administering individual tests to 40 morning and 40 evening chronotypes randomly assigned to the morning (8:00–10:00) or afternoon (13:00–15:00) sessions. The results showed a synchrony effect for fluid intelligence scores (mean of the Block Design and Forward/Backward Digit Span subtests from the Wechsler Intelligence Scale for Children–III), but not for crystallized intelligence (Vocabulary subtest from the Wechsler Intelligence Scale for Children–III).

The question remains whether results found in a laboratory setting can be observed in a school setting, which is characterized by numerous differences, such as group testing (Clarisse et al. 2010). The above-mentioned studies conducted in a school setting did not assess both fluid and crystallized intelligence directly but instead focused on grades, which can be considered a proxy for crystallized intelligence (Zhang and Ziegler 2022). In university students, who usually have more control over their class schedule than school students, morningness was related to better grades even when the timing of classes and exams was considered (Enright and Refinetti 2017). This contradicts the observation that the association between morningness and higher grades diminishes with later school day starts (Goldin et al. 2020).

Although the appearance of the synchrony effect was initially ascribed to the efficient inhibition of distractors, which seems lower at off-peak times, the core postulated mechanism driving these time-of-day differences is *circadian arousal* (May 1999). Field studies also have pointed to the potential effects of a clash between an evening chronotype and early school day starts, potentially resulting in sleep deprivation and morning academic activity that takes place shortly after, or even during, their biological night (Goldin et al. 2020). At the subjective level, effects of the above-mentioned factors can be perceived as a subjective state of sleepiness–alertness, although proof of their associations with cognitive performance in complex tasks is limited (Schmidt et al. 2007).

To sum up, although laboratory research suggests the existence of a synchrony effect for fluid, but not crystallized intelligence, studies in school settings also suggest a possible synchrony effect for crystallized intelligence as indicated by school grades. Given that school grades are influenced by multiple noncognitive factors, such as teacher expectations and student motivations (Boser et al. 2014; Mouratidis et al. 2021), we aimed to apply direct measures of crystallized and fluid intelligence to shed more light on the associations among chronotype, time of day, and intellectual performance. In line with Goldstein et al.’s (2007) study, which adopted testing times that mimicked the average school schedule, and studies conducted in real school settings (Enright and Refinetti 2017; van der Vinne et al. 2015), we were interested in performance during the real first morning versus afternoon classes, controlling for consciously available markers of circadian/sleep effects.

## 2. Method

### 2.1. Measures

#### 2.1.1. Chronotype

We used the Munich Chronotype Questionnaire (MCTQ; Jankowski 2015; Roenneberg et al. 2003) to assess chronotype. The MCTQ is a self-report instrument composed of questions about the respondent’s current sleep behavior (e.g., “I wake up at …”) separately on weekdays and free days. We used mid-sleep on free days sleep-corrected (MSFsc) as an indicator of chronotype (Roenneberg et al. 2004). MSFsc is the halfway point between sleep onset and wake-up time shifted back proportionally to the time people sleep off the sleep loss they have accumulated during workdays. MSFsc is expressed in local time, and higher values indicate eveningness. Cronbach alpha for the MCTQ based on six sleep timing indicators (bedtime, sleep onset, wake-up time, for weekdays and free days) was .80 in the current sample.

#### 2.1.2. Sleepiness–Alertness

We measured sleepiness–alertness using the Energetic Arousal subtest of the UWIST (University of Wales Institute of Science and Technology) Mood Adjective Check List (UMACL; Matthews et al. 1990), the Karolinska Sleepiness Scale (Åkerstedt and Gillberg 1990), and a visual analogue scale (Gillberg et al. 1994).

- The Energetic Arousal subscale of the UMACL comprises eight items with four response options in a Likert-type format. The total score, which is a sum of responses to the eight items, represents the participant’s level on the energetic–tired dimension. Cronbach’s α in the current sample for this scale was .88.
- The Karolinska Sleepiness Scale is a single-item self-report with nine response options ranging from *extremely alert* to *extremely sleepy, can’t keep awake*.
- A visual analogue scale asks participants to mark their position on a continuum ranging from *very sleepy* to *very alert*, with the outcome score being the distance from one edge of the dimension expressed in centimeters.

Correlations between the three measurements in the current sample were very high, ranging from .76 to .80, with Cronbach alpha = .77 in this sample; therefore, we used a composite score for sleepiness–alertness in subsequent analyses. For the composite score, a regression factor score for the first unrotated factor was derived from factor analysis (principal axis factoring), with higher values indicating alertness and lower values denoting sleepiness.

#### 2.1.3. Fluid Intelligence

We used the Culture Fair Intelligence Test, Scale 3 (CFT3; Cattell 1961; Matczak and Martowska 2013) to assess fluid intelligence. The CFT3 is composed of four nonverbal subtests (50 tasks in total). The first subtest (13 tasks) asks the testee to pick an abstract figure from among six options that best fits a series of three other figures. The second subtest (14 tasks) asks the testee to identify two figures that differ from others in a five-figure set. The third subtest (13 tasks) asks the testee to pick a figure that completes a matrix of figures whose subsequent elements differ one from another according to two rules. The fourth subtest (10 tasks) asks the testee to pick figure-dot arrangement from among five options that replicate the model figure-dot composition. Correct responses across all subtests sum up to the total score (50 points maximum), with higher values indicating higher intellectual performance. Internal consistency reliability as indicated by Spearman-Brown coefficient (odd-even split) was .75 in the current sample.

#### 2.1.4. Crystallized Intelligence

We used the Word Comprehension Test–Standard Form (Matczak et al. 2012) to assess crystallized intelligence. The test is composed of 32 tasks with increasing difficulty; on every task, the testee is requested to pick a word from among five provided that is synonymous with a stimulus word. The total score is a sum of correct responses, and greater values indicate higher intellectual performance. Internal consistency reliability as indicated by Spearman-Brown coefficient (odd-even split) was .71 in the current sample.

### 2.2. Procedure

In this cross-sectional study, time of day was a between-subjects factor, and measurements were administered in class rooms during scheduled classes. Morning measurements were administered during the first class, starting at 8:00, to a sample of 92 students. Afternoon measurements were administered during the seventh class, starting between 13:00 and 13:40, from a sample composed of 106 students. In total, 10 groups of students underwent testing, five groups in the morning and five in the afternoon. Measurement started with the sleepiness–alertness questionnaires, followed by the MCTQ, CFT3, and the Word Comprehension Test. The study was anonymous (participants did not put their names on questionnaires), voluntary, and without any remuneration. Among the students who were approached, one declined to participate. The study was conducted by an experimenter who did not belong to the school staff.

### 2.3. Participants

The sample was composed of students of the last grade attending two high schools. In total, 198 students (122 females, 76 males) between ages 17 and 20 years (*M* = 18.16, *SD* = .40) participated in the study. Using the quartile cutoff values of MSFsc based on its distribution in the total sample (evening types top quartile MSFsc > 5:30; morning types bottom quartile MSFsc < 3:54), in the morning class there were 20 evening types, 45 neither types, and 27 morning types, and in the afternoon class there were 29 evening types, 55 neither types, and 22 morning types.

### 2.4. Statistical Analyses

Regarding statistical analyses, we first computed the means and standard deviations for study variables in the total sample and separately in morning and afternoon hours, and then we compared times of the day using independent-samples *t* tests with Cohen’s *d* effect size measures. Next, we computed Pearson correlations between the study variables. Finally, we applied multiple regressions with time of day, MSFsc, and the MSFsc × Time of Day interaction term entered as predictors. MSFsc was standardized to a *z* score, and in such form was used for the calculation of interaction term. Sleepiness–alertness, fluid intelligence, and crystallized intelligence were the outcome variables in the regression models. A level of *p* < .05 (two-tailed) was considered statistically significant. Analyses were performed using IBM SPSS Statistics (Version 28).

## 3. Results

A series of independent-samples *t* tests revealed that there were no statistically significant differences and only trivial effect sizes between morning versus afternoon measurement in the study variables. The difference between morning and afternoon classes in MSFsc was also statistically non-significant with a trivial effect size that just about approached a small effect, with afternoon classes being more evening oriented (Table 1). Pearson correlations indicated that in the sample as a whole, morningness was related to higher performance on the tests measuring crystallized intelligence but was unrelated to the indicator of fluid intelligence and sleepiness–alertness (Table 2). Furthermore, students who performed better on measures of fluid intelligence also performed better on measures of crystallized intelligence. Sleepiness–alertness was unrelated to both types of intelligence (Table 2).

**Figure 1:**
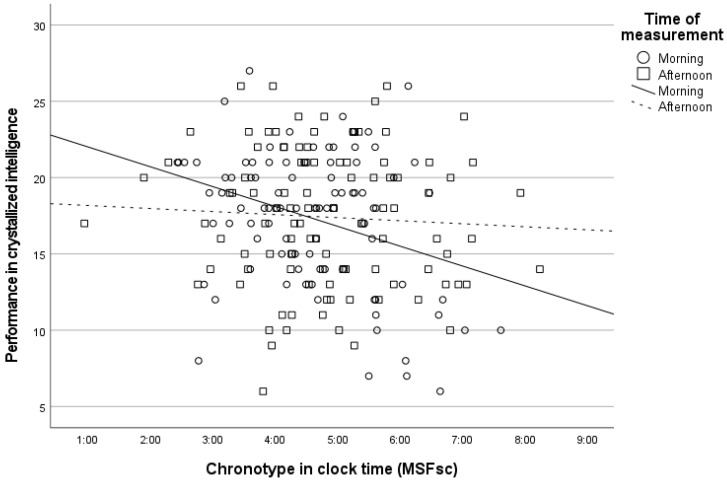
Association between chronotype (MSFsc—mid-sleep on free days, sleep-corrected) and performance on the test measuring crystallized intelligence during a morning versus an afternoon class.

Multiple linear regressions showed that the model using time of day, chronotype, and their interaction as predictors was statistically significant in predicting crystallized intelligence, but not fluid intelligence or sleepiness–alertness (Table 3). Better performance on measures of crystallized intelligence was fostered by morningness and its interaction with time of day. An analysis of this interaction (Figure 1) showed that, during the morning class, morning chronotypes performed largely better than evening chronotypes on the crystallized intelligence test (*R*^2^ = .11, *p* < .01, Cohen’s *d* = .94), whereas during the afternoon class there was no association between chronotype and crystallized intelligence (*R*^2^ = .00, *p* = .57, Cohen’s *d* = .11). This association resulted from moderately worse performance on the test of crystallized intelligence during the morning class (*M* = 14.10, *SD* = 5.53) compared with the afternoon class (*M* = 17.45, *SD* = 4.29, *p* < .05, Cohen’s *d* = .69) in the evening chronotypes, given that the performance of the morning chronotypes remained similar across morning (*M* = 18.58, *SD* = 4.05) and afternoon classes (*M* = 17.95, *SD* = 4.57, *p* = .63, Cohen’s *d* = .15).

While the overall model for fluid intelligence was statistically non-significant, an interpretation of effect sizes indicates that during the morning class, morning chronotypes performed moderately better than evening chronotypes on the fluid intelligence test (Cohen’s *d* = .71), whereas during the afternoon class the difference between chronotypes was negligible (Cohen’s *d* = .04). Interestingly, in the overall model, association between chronotype and fluid intelligence reached statistical significance indicating a possibility of suppression effect of time of day, nevertheless, we abandon interpretation of this effect due to statistical non-significance of the overall model. These effects were independent of sleepiness–alertness levels, because controlling for this variable did not change the main outcomes (Table 4).

## 4. Discussion

In this study, we aimed to verify the effects of chronotype and time of day on intellectual performance in the school setting. The results showed a synchrony effect for crystallized intelligence but not for fluid intelligence. The synchrony effect emerged from morning chronotypes exhibiting stable efficiency across morning and afternoon classes and evening chronotypes performing worse during morning classes and improving during afternoon classes. As a result, morning chronotypes outperformed evening chronotypes during morning hours, whereas in the afternoon differences between chronotypes disappeared in regard to performance on measures of crystallized intelligence. These results were independent from students’ alertness–sleepiness levels. In the following paragraphs, we discuss these outcomes in more detail.

According to the synchrony effect, people show higher performance at times that better match their individual preferences for the time of day (Hahn et al. 2012). The results of the current study, indicating an existence of the synchrony effect between time of day and chronotype for crystallized intelligence, is in line with those of previous studies on academic performance. To be specific, studies on adolescents attending morning classes have found that morning chronotypes showed better academic performance than evening chronotypes (Escribano et al. 2012; Itzek-Greulich et al. 2016). However, when adolescents were evaluated in the afternoon school shift, chronotype was not associated with academic performance, suggesting that the afternoon shift cancels out the morning handicap of evening adolescents (Arrona-Palacios and Díaz-Morales 2018). This result is corroborated by our findings, which were based on a direct measure of crystallized intelligence and was confirmed by Goldin et al. (2020), who found that, in the case of morning classes, early chronotypes performed better than late chronotypes in all school subjects, but this effect vanished for students who attended school in the afternoon, with the evening chronotypes being the ones who most benefited from evening classes. Taken together, both our results and those of Goldin et al. (2020) suggest that worse school performance of evening chronotypes during morning classes may stem more from problems with recalling declarative knowledge than compromised learning capacities, because the content of the word comprehension test that we used as a measure of crystallized intelligence did not specifically refer to the teaching program of high schools in Poland.

Our results revealed neither an effect of eveningness nor a synchrony effect on fluid intelligence. These results do not support observations of evening chronotypes exhibiting higher performance on tasks related to fluid intelligence that have been made in adults in some studies (Kanazawa and Perina 2009; Preckel et al. 2011). At the same time, we must acknowledge that studies of adults have shown inconsistent results (e.g., Zajenkowski et al. 2019). Similarly, studies of adolescents are not unambiguous. For instance, one study of adolescents who attended high school with a morning schedule reported that evening types obtained lower school grades, but they scored higher on measures of inductive reasoning compared with morning types (Díaz-Morales and Escribano 2013), but in another study the effect was only marginal, with a negligible correlation of −.07 (Díaz-Morales and Escribano 2015).

The inconsistency between the various studies that have analyzed associations between eveningness or the synchrony effect and performance on tasks engaging fluid intelligence is likely a resultant of weak effect size. In addition, in the current study the bivariate association between chronotype and fluid intelligence, as indicated by a Pearson correlation, was too weak to reach statistical significance. While we did not conduct an a priori power analysis, post hoc sample size calculations indicated that we would need at least 296 subjects to allow the association to reach statistical significance. In fact, our sample size allowed us to detect correlations of at least .14 at the commonly accepted *p* level, which anyways can be classified as a small correlation (medium > .30; Cohen 1992). Similarly, the regression model with fluid intelligence as the outcome did not reach statistical significance, which could have happened if there were at least 275 subjects. In the meantime, our sample size allowed statistical significance to be reached by models that explained at least 4% of the variance in the outcome, which still is considered a small effect (medium > 13%; Cohen 1992).

The idea of a synchrony effect can be valid (i.e., exert stronger effects) across other performance domains, populations, or test settings. For instance, in a visual search, as well as in a task involving logical, spatial, and mathematical reasoning, the best performance of university students tested in laboratory conditions was recorded during their optimal times of day according to their chronotype (Natale et al. 2003). Results from laboratory studies, however, may not always be easily generalized to more natural conditions because different situations, such as individual versus group testing, can unpredictably influence one’s daily performance (Clarisse et al. 2010).

In previous studies academic performance has often been measured by grade point average or self-reported achievement (Randler and Frech 2009). In this context, one of the advantages of the present study was our use of a standardized measure of crystallized intelligence. The main limitation, on the other hand, was a restricted sample size that made weak effects less likely to reach statistical significance.

The obtained results suggest that individual differences between chronotypes may be important for tasks that involve crystallized intelligence, especially when completed during morning classes, but not afternoon ones, and that performance across school days may be more dependent on time of day in later chronotypes. Results from the current study regarding both types of intelligence were independent of sleepiness–alertness levels, supporting observations that performance in complex tasks does not rely on circadian rhythmicity in subjective self-report states (Schmidt et al. 2007).

## Figures and Tables

**Table 1:** Descriptive statistics of the study variables for the total sample and morning versus afternoon classes (no significant differences were noted).

	Total Sample		Morning Class		Afternoon Class		Morning vs. Afternoon
	*M*	*SD*	*M*	*SD*	*M*	*SD*	Cohen’s *d*
MSFsc	4:42	1:12	4:35	1:07	4:48	1:16	.18
Fluid intelligence	3.78	6.23	31.02	6.66	3.57	5.85	.07
Crystallized intelligence	17.35	4.48	17.27	4.50	17.42	4.48	.03
Alertness	.00	1.00	.02	.99	−.02	.94	.05

Note. MSFsc—mid sleep on free days sleep-corrected.

**Table 2:** Pearson correlations with 95% confidence intervals between study variables.

	Fluid Intelligence	Crystallized Intelligence	Alertness
MSFsc	−.11 [−.23, .00]	−.17 * [−.30, −.04]	−.01 [−.16, .14]
Fluid intelligence		.39 *** [.26, .49]	−.05 [−.21, .09]
Crystallized intelligence			−.13 [−.28, .00]

Note. * *p* < .05; *** *p* < .001; MSFsc—mid sleep on free days sleep-corrected.

**Table 3:** Results of linear regressions with two types of intelligence and alertness as outcomes.

	Fluid Intelligence		Crystallized Intelligence		Alertness	
Predictor	β [CI]	*R* ^2^	β [CI]	*R* ^2^	β [CI]	*R* ^2^
Time of day	−.05 [−.18, .09]	.03	.03 [−.11, .17]	.05 *	−.02 [−.16, 12]	.01
MSFsc	−.25 * [−.46, −.03]		−.35 ** [−.57, −.13]		−.11 [−.34, .11]	
Time of day×MSFsc	.18 [−.04, .39]		.23 * [.01, .45]		.13 [−.09, .36]	

Note. * *p* < .05; ** *p* < .01; CI—95% confidence interval; Time of day is coded 0 = morning, 1 = afternoon; MSFsc—mid sleep on free days sleep-corrected.

**Table 4:** Results of a linear regression predicting intelligence after controlling for sleepiness–alertness.

	Fluid Intelligence		Crystallized Intelligence	
Predictor	β [CI]	*R* ^2^	β [CI]	*R* ^2^
Time of day	−.05 [−.18, .09]	.03	.05 [−.11, .16]	.07 **
Alertness	−.07 [−.21, .07]		−.15 * [−.29, −.01]	
MSFsc	−.25 * [−.47, −.04]		−.37 ** [−.59, −.15]	
Time of day × MSFsc	.19 [−.03, .40]		.25 * [.03, .47]	

Note. * *p* < .05, ** *p* < .01; CI—95% confidence interval; Time of day is coded 0 = morning, 1 = afternoon; MSFsc—mid sleep on free days sleep-corrected.

## Data Availability

The data presented in this study are available on request from the corresponding author.
